# Metabolic Landscape and Emerging Therapeutic Potential in Pediatric and Adult Gliomas

**DOI:** 10.3390/ijms27062720

**Published:** 2026-03-17

**Authors:** Cayley S. Brock, Lam Nguyen, Curtis Pattillo, Cheyenne J. Ahamed, Keisaku Sato, Kevin K. Kumar

**Affiliations:** 1 Department of Neurosurgery, Dell Medical School, The University of Texas at Austin, Austin, TX 78701, USA; cb54883@my.utexas.edu (C.S.B.); lgn326@my.utexas.edu (L.N.); curtis.pattillo@utexas.edu (C.P.); cheyenne.ahamed@austin.utexas.edu (C.J.A.); keisaku.sato@austin.utexas.edu (K.S.); 2 UT Health Austin Pediatric Neurosciences, Dell Children’s Medical Center, Austin, TX 78701, USA

**Keywords:** glioma metabolism, metabolic heterogeneity, metabolomics, cancer metabolism, metabolic dysregulation

## Abstract

The underlying metabolism of tumor cells in gliomas has become an area of focus secondary to the difficulties in diagnosis and treatment of these tumors. Heterogeneity in both molecular and phenotypic features of tumor cells in pediatric and adult gliomas presents a significant barrier to traditional treatment options such as radiotherapy and chemotherapy. Low-grade gliomas in pediatric and adult populations have relatively high survival rates, while high-grade gliomas have no effective treatments. Recent advancements in metabolomic techniques have uncovered key metabolic abnormalities, such as increased glutamine and creatinine in invasive edge cells and increased purines in viable tumor cells, distinguishing tumor cells in gliomas. Spatial metabolic heterogeneity and metabolic plasticity enable gliomas to adapt to diverse microenvironments and oxidative stress, necessitating precision medicine approaches that target subtype-specific metabolic vulnerabilities. Further, gliomas are characterized by high intratumoral heterogeneity, with metabolic distinctions between core, edge, viable, and necrotic regions. Altered metabolism of tumor cells has an impact on cells within the tumor microenvironment, resulting in a dysfunctional phenotypic state in resident cells. These metabolic abnormalities differentiate tumor cells from the surrounding microenvironment. Enhanced understanding of the metabolic abnormalities in gliomas could inform targeted therapies, increasing therapeutic response in patients. This review synthesizes emerging evidence on intratumoral and intertumoral heterogeneity in gliomas, highlights the role of tumor-immune cell crosstalk in shaping the metabolic landscape, and discusses how these vulnerabilities may be exploited to develop novel therapies.

## 1. Introduction

Central nervous system (CNS) tumors are the most common solid tumors in pediatric patients, accounting for approximately 21% of pediatric cancers in the United States and serving as a significant cause of mortality [1]. The survival rate of pediatric gliomas is dependent on the tumor grade; lower grades (I–II) indicate less cellular abnormality while higher grades (III–IV) reflect more aggressive cellular changes when observed under the microscope [1]. Pediatric low-grade gliomas (pLGGs) generally have a good prognosis, with a 20-year overall survival (OS) rate of approximately 87% [1]. Because pLGGs typically have clear boundaries, they are often treated with surgical resection, which can be curative depending on its extent [2]. Pediatric high-grade gliomas (pHGGs) are typically diffuse and infiltrative, which makes surgical removal challenging [1,3]. They have a poor prognosis, with a 5-year OS rate of less than 20% even with traditional treatments, such as radiotherapy and chemotherapy [2]. 

Treatment strategies and OS depend largely on glioma subtype, and it is important to understand and accurately categorize the underlying tumor architecture. Historically, pediatric gliomas have been classified based on histopathological features; however, this approach presented significant challenges given the diverse and overlapping morphologic characteristics among gliomas [4]. As a result, there has been an emphasis in the modern literature on the use of molecular markers to classify gliomas, as reflected in the fifth edition of the Classification of Tumors of the CNS released by the World Health Organization (WHO) in 2021 [4]. The revised classification of gliomas represents a shift toward molecularly informed diagnosis, emphasizing that glioma biology is defined by tumor entity, molecular driver, and microenvironmental context [4]. This framework also clarifies important distinctions between pediatric-type and adult-type gliomas while avoiding broad age-based generalizations [4].

The metabolism of tumor cells can be studied as a method of better understanding the molecular nature of gliomas. Metabolic reprogramming plays an important role in the enhanced growth and proliferation of tumor cells during glioma development [5]. One of the hallmark features of glioma metabolism is the Warburg effect, where glioma cells exhibit a stronger preference for glycolysis as their primary mode of energy production even in an aerobic environment [6]. Alongside glycolysis, glioma cells heavily rely on glutamine metabolism, as glutamine supports energy production and biosynthesis by acting as a substrate for the tricarboxylic acid cycle (TCA) [6]. Glioma cells also exhibit abnormalities in lipid metabolism [7]. Specifically, increased fatty acid oxidation and lipid synthesis aid tumor proliferation by increasing energy supply and biosynthesis [7]. Metabolism has been identified as a key component of glioma molecular architecture resulting in increasing use of metabolomics as a tool for investigating metabolic reprogramming and molecular makeup within tumors [5]. Metabolomic tools, such as nuclear magnetic resonance (NMR) spectroscopy and mass spectrometry (MS), can detect and identify key metabolites in tumor samples, offering valuable insights into reprogrammed metabolic pathways and potential therapeutic targets [5].

While interest in targeting aberrant metabolism in gliomas has grown, clinical trials targeting metabolic pathways remain limited compared to other approaches, such as immunotherapy [8]. This disparity stems largely from the heterogeneous nature of metabolic pathways across different glioma subtypes [8]. A contributing factor to this problem is the glioma tumor microenvironment (TME). The TME is composed of various cellular and noncellular components that interact with tumor cells, facilitating progression, immune evasion, and therapy resistance [8,9]. These components include microglia, T cells, natural killer cells, extracellular matrix proteins, and neural cells such as neurons and astrocytes [9]. The presence and proportion of these elements vary significantly between glioma subtypes, resulting in intertumoral heterogeneity [9,10,11]. Additionally, variation in the spatial distribution and composition of different immune cell types and their interactions within distinct regions of a single tumor contributes to intratumoral heterogeneity [10,12]. A deeper understanding of the intersection between abnormal metabolism in glioma cells, the TME, and the associated heterogeneity may accelerate progress in advancing more therapies to clinical trials.

Studies included in this review were identified through a literature search of PubMed. A combination of key words were used to identify relevant publications, including “pediatric glioma”, “metabolism”, “glycolytic metabolism”, “pediatric glioma lipid metabolism”, “intertumoral heterogeneity”, “intratumoral heterogeneity”, “glioma and neuron interactions”, “glioma and macrophage interactions”, “glioma stem cells”, “metabolism inhibition”. These terms were combined using “AND” and “OR” operators as appropriate. Additional relevant articles were identified through reference lists of reviewed papers. Inclusion criteria were English language, glioma-specific, clinical trials, experimental studies, or literature reviews. Exclusion criteria were case reports, editorials, and metabolomics studies on non-glioma neoplasms. The initial search period was 2016–2026. However, older studies were included when necessary due to limited literature in selected areas of glioma metabolism.

This review aims to address the gaps in developing cellular therapeutics that target aberrant metabolic activity in pediatric and adult gliomas. Specifically, we explore how metabolomics can enhance our understanding of intratumoral and intertumoral heterogeneity, metabolic interactions between normal cells and the TME. We also discuss how these insights can help researchers identify glioma-specific metabolic abnormalities to guide the development of personalized therapies.

## 2. Heterogeneity in Pediatric and Adult Gliomas

### 2.1. Intratumoral Heterogeneity

Metabolomic analysis has provided important insights into how intratumoral heterogeneity affects the underlying biology of pediatric and adult gliomas [13]. In grade 3 medulloblastoma, there are subclonal populations that demonstrate amplification of the myelocytomatosis (*MYC*) proto-oncogene, a gene often amplified in pHGGs that promotes uncontrolled cell growth and prevents cell differentiation [14]. Non-*MYC*-driven cells take up extracellular lactate dehydrogenase A (LDHA), an enzyme that catalyzes the conversion of pyruvate to lactate, released from *MYC*-driven cells [14]. Liquid chromatography-mass spectrometry (LC-MS) analysis demonstrated that internalized LDHA remained enzymatically active and promoted the Warburg effect in recipient non-*MYC*-driven cells, making these cells more invasive [14].

**Figure 1:**
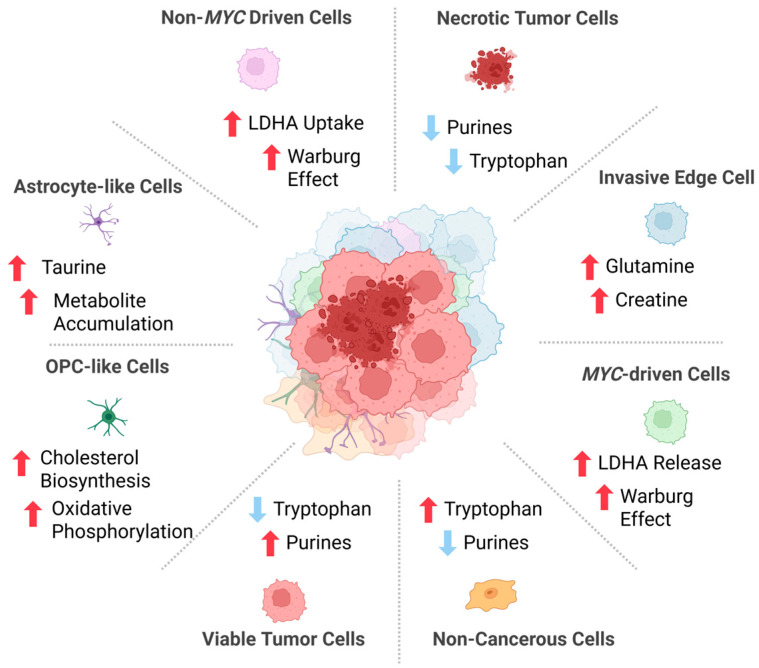
Intratumoral heterogeneity in metabolites of the glioma. Metabolomic analysis has revealed key metabolite differences between cells within a glioma, providing insight into underlying tumor biology. Subclonal populations exhibit diversity in expression of the myelocytomatosis (*MYC)* proto-oncogene, with *MYC*-driven cells promoting uncontrolled cell proliferation and inducing excessive lactate dehydrogenase A (LDHA) uptake in surrounding non-*MYC* cells, contributing to the Warburg effect. Subclonal diversity exists between viable, core, edge, and non-cancerous regions. Viable tumor cells are marked by increased purine levels, indicating higher levels of DNA repair. Necrotic tumor cells exhibit lower levels of purine and tryptophan, indicating lower metabolic levels and decreased DNA repair. Edge cells exhibit high levels of glutamine and creatine, indicating elevated metabolism. Non-cancerous cells exhibit increased levels of tryptophan, as non-cancerous cells do not shunt tryptophan to kynurenine, an abnormal metabolic trait of tumor cells. Oligodendrocyte precursor (OPC)-like stem cells demonstrate increased cholesterol biosynthesis and oxidative phosphorylation, likely to support increased proliferation and invasion. Astrocyte-like cells exhibit increased metabolite accumulation due to their role in metabolic homeostasis of the brain. *MYC*, myelocytomatosis; LDHA, lactate dehydrogenase A; OPC, oligodendrocyte precursor-like cells. Created in BioRender. Kumar, K. (2026) (https://BioRender.com/r9tc9em, accessed on 14 March 2026).

Other high-grade gliomas (HGGs) exhibit subclonal diversity in the form of viable, necrotic, and non-cancerous regions [15] (Figure 1). MS analysis of glioblastoma (GBM) demonstrated that the viable regions exhibited increased levels of purines and purine derivatives such as xanthine and 8-hydroxy-7-methylguanine [15]. Non-cancerous cells were demonstrated to have decreased levels of purines and increased levels of tryptophan [15]. Purines have been shown to stimulate DNA repair and promote radiotherapy resistance in tumor cells [16]. Intratumoral heterogeneity also exists between the core and edge regions of gliomas [17,18]. LC-MS analysis of core and edge regions from 27 patient samples indicated that edge regions had elevated levels of metabolites such as glutamine, creatine, and nicotinamide [17]. These metabolites are indicators of elevated metabolism and help explain the increased invasiveness of glioma edges and their metabolic role in glioma progression [17]. Furthermore, glioma cells that are positive for O^6^-methylguanine DNA methyltransferase, a marker for improved OS, had higher hydroxyhexanoic carnitine and lower spermine levels in the core regions and higher itaconic acid and pantothenic acid levels in the edge regions [17]. Another study found that edge regions demonstrated enhanced purine and pyrimidine metabolism due to elevated levels of purines (xanthine and hypoxanthine) and pyrimidines (uridine and deoxyuridine) compared to core regions [18]. The enhanced purine and pyrimidine metabolism suggests enhanced metabolic activity in the edge region, indicative of a metabolic role in glioma invasion [17].

Diffuse midline glioma (DMG), another pHGG, demonstrates diverse subclonal populations: less-differentiated oligodendrocyte precursor (OPC)-like stem cells and more differentiated astrocyte-like cells [19]. OPC-like cells, which are associated with a poorer prognosis, demonstrated upregulation of cholesterol biosynthesis and mitochondrial oxidative phosphorylation [19]. Differentiated glioma cells exhibited a ten-fold increase in taurine, which has been shown to induce differentiation of OPC-like cells [19]. Figure 1 summarizes the intratumoral heterogeneity of metabolites and component cells characteristic of glioma biology.

### 2.2. Intertumoral Heterogeneity in Gliomas

#### 2.2.1. Metabolic Signatures Shaped by Gene Expression

Mutations in *isocitrate dehydrogenase* (*IDH*) have been linked to heterogeneity in metabolism between different glioma subtypes [20,21,22]. *IDH* catalyzes the decarboxylation of isocitrate into α-ketoglutarate (α-KG) in the TCA cycle [20,21]. Mutations in *IDH1* and *IDH2* result in the conversion of α-KG to the oncometabolite D-2-hydroxyglutarate (D2-HG) [20]. D2-HG then disrupts enzymes that aid in DNA and histone methylation, contributing to epigenetic dysregulation and aberrant proliferation [20]. The prevalence of *IDH* mutations in the pediatric population ranges from 1% to 50%, with *IDH1* mutations being more common than *IDH2* mutations [20]. *IDH1/2*-mutant gliomas are predominantly adult-type diffuse gliomas. In pediatric populations, *IDH* mutations are rare overall and primarily observed in older children, adolescents, and adolescent/young adult glioma cohorts at a prevalence of 5–15% [20].

Metabolomic analysis has revealed important differences in metabolism between wild type (WT) and *IDH*-mutant glioma cells [23] (Table 1). *IDH*-mutant lesions contain lower levels of glutamate and gamma-aminobutyric acid (GABA) compared to WT gliomas [23]. GABA production is strongly correlated to the available amounts of glutamate and glutamine, potentially indicating dysregulation in glutamate and glutamine metabolism [23]. Furthermore, *IDH*-mutant HGGs (grades III and IV) demonstrated elevated levels of choline compared to *IDH*-mutant low-grade gliomas (LGGs) (grades I and II), indicating enhanced phospholipid metabolism through increased cell membrane turnover and proliferation [23,24]. *IDH1*-mutant samples contained significantly less adenosine triphosphate (ATP), indicating impaired energy production [21]. These results were complemented by findings that carnitine and acylcarnitine levels were significantly reduced, leading to suppression of β-oxidation and further decreased ATP production [21]. The reduction in energy metabolism may be related to the more favorable prognosis associated with *IDH1*-mutant gliomas, while the significant difference in carnitine levels may represent a useful biomarker for these tumors [21,25]. These results suggest that *IDH1* mutations can serve as both a diagnostic and prognostic biomarker in gliomas, as they are used in both molecular classification of gliomas and are associated with improved OS compared to *IDH*-WT gliomas [21,25]. Lipid-based metabolomics has revealed that several lipid synthesis precursors, such as glycerol, glycerol-3-phosphate, ethanolamine, and inositol, are elevated in *IDH*-mutant gliomas compared to *IDH*-WT gliomas, demonstrating enhanced lipid synthesis in *IDH*-mutant gliomas [26].

Higher D2-HG levels secondary to *IDH1* mutations result in hyperactivation of the mTOR signaling pathways in neurons, leading to increased neuronal excitability and promotion of seizure activity in the brain [22]. Neurons exposed to D2-HG also exhibited increased LDHA levels, representing a shift towards glycolytic metabolism [27]. The functional roles of LDHA in *IDH1*-mutant gliomas are still undefined, as another study demonstrated that LDHA is downregulated in *IDH*-mutant glioma samples due to increased LDHA promoter methylation caused by D2-HG [28].

**Table 1:** Key molecular drivers of associated glioma subtype and typical tissue sampling material (tissue biopsy, cerebrospinal fluid (CSF), or plasma).

WHO-Aligned Glioma Entity	Molecular Driver	Tissue Sampling	Reference
Pediatric-type low grade glioma	*KIAA1549-BRAF*, *BRAF V600E*	Biopsy	[29]
Pediatric-type high grade glioma, H3-WT and *IDH*-WT	*TP53*, *ATRX*, *PDGFRA, H3G34*	Biopsy, CSF	[29,30]
DMG H3K27M-altered	H3K27M, *TP53*, *ACVR1*, *PDGFRA*, *EGFR*	Biopsy, CSF	[29,30]
Diffuse hemispheric glioma, H3G34-mutant	H3G34, *TP53*, *ATRX*, *PDGFRA*	Biopsy, CSF	[29,30]
Adult-type diffuse astrocytoma, *IDH*-mutant	*IDH1/2*, p53, ATRX	Biopsy	[29,31]
Oligodendroglioma, *IDH*-mutant and 1p/19q-codeleted	*IDH1/2*, 1p/19q co-deletion	Biopsy	[29]
GBM, *IDH*-WT	TERT promoter, chromosomes 7/10, *IDH1/2* EGFR	Biopsy, CSF	[29,31,32]

#### 2.2.2. Lipid Metabolism

Abnormal lipid metabolism is one of the most common metabolic pathway deviations found in glioma cells [33,34]. Lipids help tumor cells sustain their high rates of proliferation and invasion by providing a source for energy, cellular membranes, and signaling molecules [33,34]. Many lipids, such as oxysterol and cholesterol, also contribute to upstream gene regulation by interacting with sterol-regulating elements, affecting lipogenesis in tumor progression [33,34].

MS analysis of eight pediatric medulloblastoma and three pineoblastoma biopsy specimens revealed that certain types of lipids could help differentiate medulloblastomas from pineoblastoma, as these two glioma subtypes share many histopathological features [35,36]. Medulloblastomas are characterized by higher levels of glycerophosphoglycerol and glycerophosphocholine, while pineoblastomas contain higher levels of sphingolipids [35,36]. Medulloblastomas, along with atypical teratoid rhabdoid tumors, contained elevated lipid concentrations when compared to pilocytic astrocytomas and ependymomas. This finding suggests that the former two glioma subtypes have higher metabolic activity and membrane turnover [37]. Differences in lipid metabolism have also been identified between different glioma grades [38,39]. For example, lipidomic analysis of 76 glioma specimens of varying grades demonstrated that lysophosphatidylethanolamine (LPE), a cell membrane lipid mediator of metabolism, was found in lower abundance in HGGs compared to LGGs, suggesting that LPE may serve as a potential prognostic biomarker for determining tumor grade [38]. Furthermore, pHGGs demonstrated a higher choline/creatine ratio and lower N-acetylaspartate (NAA)/creatine and NAA/choline ratios compared to pLGGs in ^1^H magnetic resonance spectroscopy (MRS) analysis of 209 pathologically confirmed pediatric glioma samples [39]. This finding suggests that lower NAA metabolite ratios correlate with more severe tumor infiltration of brain tissue [39]. Furthermore, *IDH1*-mutant glioma cells have higher levels of saturated and monounsaturated fatty acids in the endoplasmic reticulum, leading to larger amounts of monounsaturated fatty acid-containing phosphatidylethanolamines and phosphatidylcholines and relative deficiency of polyunsaturated fatty acids [40]. Conversely, these saturated fatty acid and monounsaturated fatty acid phospholipids are reduced in the Golgi [40]. This study demonstrates a distinct lipid profile for *IDH1*-mutant gliomas and evidence of lipid heterogeneity among organelles within glioma cells [40].

Lipid-based metabolomics has also identified age-associated differences across selected glioma cohorts and models [41,42]. In one comparative analysis, adult glioma samples demonstrated higher levels of acylcarnitines, a byproduct of fatty acid oxidation, than pediatric glioma samples [41]. In the pediatric glioma cohort of the comparative analysis, acylcarnitine levels did not significantly differ across subtypes, suggesting less reliance on fatty acid oxidation in specific pediatric glioma samples [41]. Pre-clinical studies of cultured glioma cell lines have shown that cholesterol depletion results in greater growth reduction in pediatric models compared to adult models, suggesting that cholesterol metabolism may be a potential metabolic therapy target [42]. Metabolomic analyses have also revealed distinct lipid profiles between glioma tissue and normal brain parenchyma [43]. Phospholipids such as phosphatidylserine and sulfatide are elevated in gray and white matter, whereas phosphatidylcholine and phosphatidylinositol are elevated in gliomas [43].

#### 2.2.3. Glucose and Amino Acid Metabolism

Glycolysis not only provides energy but also produces intermediates used in anabolic processes and cell proliferation [44]. Glioma cells also demonstrate the Warburg effect and depend heavily on glutamine to support biosynthesis of key macromolecules [44,45,46]. Differences in glucose metabolism have been observed between different glioma grades [47]. One study found that lactate and alanine levels are significantly elevated in pHGGs, especially in ones with the *IDH*-WT phenotype [47]. Furthermore, medulloblastoma exhibits significantly lower glucose levels compared to other cerebellar tumors, indicating increased glycolytic activity [37]. Conversely, pilocytic astrocytomas demonstrate less glycolytic activity and greater reliance on glutamine metabolism for energy production and biosynthesis [37].

Amino acids not only serve as building blocks for protein, but are also important metabolic regulators, neurotransmitters, and precursors for other metabolites [48]. Due to their importance in maintaining cellular functions, gliomas require elevated levels of amino acids for rapid proliferation [48]. Differences in amino acid metabolism serve as another important source of intertumoral heterogeneity [49]. Metabolomic analysis of amino acid levels in plasma samples demonstrates that phenylalanine, tyrosine, and tryptophan were significantly increased in medulloblastoma compared to other intracranial tumors [49]. These results suggest that abnormalities in phenylalanine, tyrosine, and tryptophan metabolism could potentially be specific to medulloblastomas [49]. ^1^H NMR Spectroscopy has revealed that HGGs exhibited higher levels of taurine, phenylalanine, and tyrosine but lower levels of glutathione in comparison to LGGs [50]. The increased taurine indicated that HGGs underwent altered osmoregulation and antioxidant responses [50]. Furthermore, the higher levels of phenylalanine and tyrosine in HGGs correlated with enhanced amino acid metabolism and biosynthesis for energy as compared to LGGs [50]. Amino acid metabolism and extracellular availability play an important role in pediatric glioma activity because of the impact of amino acids on the epigenetic landscape. In DMG H3K27M-altered tumors, glioma cells use glutamine in the microenvironment to produce α-KG, which fuels α-KG-dependent histone demethylases and maintains an open, pro-proliferative chromatin state [51]. When glutamine metabolism is limited, α-KG levels decline and chromatin shifts toward a more repressed state [51]. More recent findings suggest that hyaluronic acid, an important extracellular matrix component, modifies phenylalanine, tyrosine, tryptophan, and branched-chain amino acid pathways in gliomas under glucose-deficient conditions [52]. Consequently, hyaluronic acid alters mitochondrial respiration and the TCA cycle, improving tumor cell survival under nutrient-deficient microenvironmental conditions [52]. These findings highlight the metabolic diversity in amino acid and glucose metabolism that exist between different glioma subtypes.

## 3. Metabolic Crosstalk in the Tumor Microenvironment

### 3.1. Glioma–Immune Cell Metabolic Interactions

#### 3.1.1. T Lymphocytes

**Figure 2:**
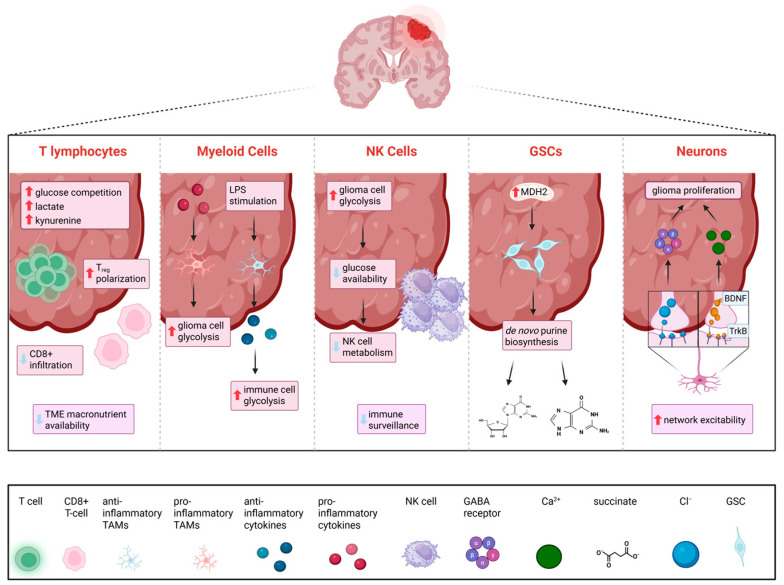
Glioma–immune cell metabolic interactions. Glioma cells communicate with immune cells within the tumor microenvironment (TME), leading to changes in metabolic states within resident immune cells. Local depletion of nutrients due to increased glioma cell metabolism results in early exhaustion of pro-inflammatory T lymphocytes and decreased CD8+ infiltration, contributing to an immunosuppressive state. Lipopolysaccharide (LPS) stimulation polarizes tumor-associated macrophages (TAMs) into a pro-inflammatory state, decreasing immune cell glycolysis rates. Pro-inflammatory cytokine secretion polarizes macrophages into an M2 phenotype, increasing glioma cell glycolysis. Restricted glucose availability due to hyperactive glycolysis in glioma cells reduces natural killer (NK) cells’ immune surveillance functions and metabolism. Glioma stem cells (GSCs), made abundant by increased malate dehydrogenase 2 (MDH2) activity, exhibit metabolic flux and increased de novo purine biosynthesis. Glioma progression is regulated in part by glioma–neuron interactions, and changes in electrochemical signaling lead to enhanced network excitability and neuronal death. Increased Cl^−^ efflux leads to gamma-aminobutyric acid (GABA) activation, and GABA is metabolized into succinate and subsequently fed into the tricarboxylic acid cycle (TCA) to fuel tumor proliferation. Neuronal activity leads to the secretion of brain-derived neurotrophic factor (BDNF), triggering calcium efflux and increased glutaminergic activity. T_reg_, regulatory T cell; TME, tumor microenvironment; LPS, lipopolysaccharide; NK, natural killer; GSC, glioma stem cell; MDH2, malate dehydrogenase 2; BDNF, brain-derived neurotrophic factor; TAMs, tumor-associated macrophages; GABA, gamma-aminobutyric acid. Created in BioRender. Kumar, K. (2026) (https://BioRender.com/p9kxc92, accessed on 14 March 2026).

T lymphocytes are an important component of the adaptive arm of the immune system. CD4+ and CD8+ T cells help eliminate pathogens and tumor cells while regulatory T cells suppress pro-inflammatory activity [53]. Studies have demonstrated that T cells in the glioma TME often exist in a functional suppressed state due to their metabolic interactions with glioma cells [53,54,55]. Because glioma cells exhibit higher rates of glucose and amino acid metabolism, there is a local depletion of these important macronutrients in the TME [53]. One study found that competition for resources leads to decreased glucose consumption and early exhaustion of energy in pro-inflammatory T cells that infiltrate the glioma TME [54] (Figure 2). This conclusion is further supported by the finding of significant downregulation of glycolysis genes in early exhausted T cells compared to naïve T cells [54]. There are several mechanisms that contribute to this immunosuppressive state. Metabolomic analysis revealed that glucose deprivation suppresses IFN-γ and CD40L and leads to decreased phosphoenolpyruvate, shifting T cells into an immunosuppressive state [55]. Furthermore, the accumulation of lactate due to elevated glycolysis can help create an immunosuppressive environment by limiting CD8+ T cell migration and infiltration into the glioma TME [53]. Similar findings are seen regarding amino acid metabolism [53,56]. Elevated tryptophan metabolism leads to accumulation of kynurenine, which has been linked to lower levels of CD8+ T cells and increased regulatory T cell polarization [56].

#### 3.1.2. Myeloid Cells: Microglia and Macrophages

Tumor-associated macrophages (TAMs) are an important class of infiltrates found in the glioma TME [57]. They consist of both bone marrow-derived macrophages and microglia, resident CNS immune cells [57]. TAMs have distinct phenotypes that result in different immune functions; they can be polarized into the proinflammatory phenotype through bacterial lipopolysaccharide (LPS) and IFN-γ or the anti-inflammatory phenotype through IL-4 and IL-13 [58]. TAM activation is accompanied by metabolic reprogramming [59]. Experiments in both human and mice microglial cells reveal that proinflammatory polarization due to LPS stimulation shifts energy metabolism from oxidative phosphorylation to glycolysis [59]. Anti-inflammatory TAMS have been found to exhibit lower levels of glycolysis than their proinflammatory counterparts and rely more on oxidative forms of metabolism [59]. Further studies have demonstrated that TAM polarization is significantly affected by glioma metabolism [60]. Lactate produced from enhanced glycolysis in gliomas upregulates H3K18La and TNF molecules, inducing anti-inflammatory polarization [60]. Another study found that TAMs in the perivascular and subarachnoid space express indoleamine 2,3-dioxygenase 1, an immunosuppressive enzyme that converts tryptophan to kynurenine, and demonstrate increased phagocytic activity and suppression of inflammatory response [61]. Furthermore, anti-inflammatory macrophages secrete IL-6, which stimulates 3-phosphoinositide-dependent protein kinase 1-mediated phosphoglycerate kinase 1 threonine 243 phosphorylation in glioma cells [62]. This mechanism enhances substrate affinity in the phosphoglycerate kinase 1 catalyzed reaction in glycolysis, ultimately enhancing glycolysis in tumor cells [62]. Similarly, anti-inflammatory macrophages produce IL-1β, which results in the phosphorylation of glycerol-3-phosphate dehydrogenase, a glycolytic enzyme, at threonine 10 in glioma cells [63]. This phosphorylation enhances substrate affinity and increases glycolysis rates in glioma cells [63].

#### 3.1.3. Natural Killer (NK) Cells

NK cells are innate immune cells that participate in immune-surveillance and help eradicate virally infected and tumor cells [64]. Since NK cells can be activated without prior sensitization, they are a potentially significant point of interest for glioma therapies [64]. Nutritionally, NK cells are in direct competition with glioma cells for resources such as glucose [65]. This glucose restriction reduces glycolysis rates in NK cells, reducing their anti-tumor functions [65]. NK cells heavily rely on glucose to perform glycolysis, oxidative phosphorylation, and immune functions such as cytokine activation and degranulation [65]. NK cell activation results in enhanced glycolysis and oxidative phosphorylation; these cells also use arginine and glutamine to support their cellular functions [65]. Metabolic flux analysis revealed that *c-MYC*, a proto-oncogene that regulates cell proliferation and apoptosis, levels are regulated by glutamine. Decreased levels of glutamine results in decreased *c-MYC* expression, leading to reduced NK cell metabolism and further inhibition of anti-tumor functions [66]. Furthermore, the production of several immunosuppressive molecules, such as adenosine, by these highly glycolytic glioma cells further suppress activation of NK cells [64].

### 3.2. Metabolic Interactions Between Gliomas, Neurons, and Glioma Stem Cells

#### 3.2.1. Glioma Stem Cells (GSC) 

Even though GSCs have been found in pHGGs, they have been understudied in the pediatric population compared to the adult population, partly due to the low incidence of pHGGs and limited amount of pHGG tissue available for experimental analysis [67]. Studies have found certain putative markers for GSCs in pHGGs, such as L1 Cell Adhesion Molecule, SOX2, and Nanog [67]. However, most studies focusing on metabolic interactions between glioma cells and GSCs are in the adult population. GSCs exhibit two distinct metabolic phenotypes: increased consumption of glucose and production of lactate (glycolysis-dominant) or increased oxygen consumption and higher ATP production (oxidative phosphorylation dominant) [68]. While both phenotypes are stable independently, the oxidative phosphorylative phenotype can flux to the glycolytic phenotype in the presence of metabolic stress, such as hypoxia, suggesting that GSCs have metabolic plasticity [68]. This metabolic plasticity capability allows GSCs to survive in stressful environments by continuing to generate ATP through glycolysis instead of succumbing to hypoxia-induced stress [68]. While metabolic plasticity is a hallmark characteristic of malignant cells, the glycolysis-oxidative phosphorylation switch in stem cells makes this population a highly aggressive and drug-resistant population of particular interest [69]. Integrative metabolomic analysis using LC-MS demonstrated that compared to differentiated glioma cells, GSCs demonstrated higher amounts of metabolites derived from glycolysis, the TCA cycle, and pyrimidine metabolism, indicating increased utilization of these metabolic pathways [70]. GSCs exhibit significant upregulation of the malate-aspartate shuttle, with malate flux regulated by malate dehydrogenase 2 (MDH2), a key mitochondrial enzyme within this pathway [70]. Notably, increased MDH2 expression has been associated with GSC abundance [70]. Additionally, GSCs have higher demands for lipids, amino acids, and other nutrients [70]. Using LC-MS and metabolic flux analysis, one study found that GSCs are often able to grow and proliferate in acidic TMEs due to de novo purine nucleotide biosynthesis [71]. This is accomplished by high utilization of carbon from glucose to produce nucleotides through the pentose phosphate pathway, which is enhanced due to overexpression of glucose-6-phosphate dehydrogenase [71].

#### 3.2.2. Neurons

Pre-clinical patient-derived xenograft studies utilizing both pediatric and adult glioma models have demonstrated that glioma–neuron interactions are bidirectional: neuronal activity increases glioma cell proliferation while glioma cells increase neuronal activity [72]. Progression and proliferation of glioma cells are regulated by glioma–neuron interactions, which include both paracrine signaling and electrochemical signaling through the α-amino-3-hydroxy-5-methyl-4-isoxazolepropionic acid (AMPA) receptor [72]. One pre-clinical study demonstrated that malignant synapses display adaptive plasticity to strengthen neuron-tumor interactions [72]. Specifically, neuronal activity triggers Brain-Derived Neurotrophic Factor (BDNF) secretion, which binds to TrkB receptors on glioma cells, amplifying glutamatergic synaptic activity and calcium signaling in glioma cells [72]. Brain slices from mice implanted with human glioma cells demonstrated that increased network excitability in regions adjacent to the tumor [73]. Increased network excitability is secondary to the aberrant glutamate released from glioma cells via the system $x_{c}^{-}$, a cystine-glutamate antiporter that mediates glutamate release from glioma cells [73]. Recent studies have illuminated other glioma–neuron interactions not related to glutamate [74]. Whole-cell patch-clamp electrophysiology, in vivo optogenetics, and patient-derived orthotopic xenograft models studies revealed that there are GABAergic synapses between neurons and H3K27M+ DMG cells [74]. Since these glioma cells express high levels of the Na-K-2Cl co-transporter, GABA activation led to efflux of Cl^−^, resulting in depolarization [74]. Gliomas reprogram this cycle by upregulating *glutamate decarboxylase 1*, resulting in the conversion of glutamine-derived glutamate into GABA [75]. GABA can be metabolized into succinate, which enters the TCA cycle and fuels tumor growth, or used to produce γ-hydroxybutyrate through a branch pathway to retain GSC properties [75]. The over-reliance on GABA to shunt succinate into the TCA cycle represents a unique metabolic vulnerability of the H3K27M+ GBM subtype. Metabolic therapeutics targeting GABA synthesis, or succinate synthesis from the catabolism of GABA, may be a promising avenue of therapeutic research for this subtype. Figure 2 summarizes the metabolic glioma–immune cell interactions within the TME that contribute to metabolic changes within gliomas.

## 4. Identification of Metabolic Abnormalities and Development of Therapies

### 4.1. Metabolic Abnormalities Targeting

Metabolomic profiling of tumor subtypes may uncover specific biomarkers to guide therapeutic development and the ability to actively monitor treatment response. Notably, metabolomics research has been used to help verify the creation of biologically accurate animal models for in vivo investigation into tumor characteristics [76]. Both in vivo ^1^H-MRS and high-resolution magic angle spinning have been used to characterize the metabolic profile of the smoothened mouse (SMO), a novel medulloblastoma mouse model characterized by mutations in the *SHH* signaling pathway [76]. The SMO tumors exhibited extremely reduced levels of NAA and elevated levels of taurine, glycine and choline-containing metabolites (CCMs)—a metabolic pattern consistent with metastatic medulloblastoma, verifying its use as a reliable preclinical model and paving the path for future studies [76].

Analyzing glioma metabolism can enable the development of targeted therapies that are uniquely situated to address the specific metabolic disturbances associated with different glioma subtypes. Cholesterol metabolism presents a promising therapeutic target in HGGs, as HGGs upregulate cholesterol levels to drive tumor proliferation by increasing expression of the low-density lipoprotein receptor (LDLR) [77]. HGGs depend on LDLR to maintain high intracellular cholesterol levels essential for rapid membrane synthesis and cell–cell signaling [77]. Immunohistochemistry performed on patient-derived tissue microarrays revealed consistently elevated LDLR expression across intratumoral regions in both pediatric-type diffuse high-grade gliomas (PDHGGs) and adult GBM, as well as across pediatric tumor grades [77]. These findings support targeting of LDLR as a therapeutic option suitable across age ranges and may help bridge current disparities between pediatric and adult glioma therapy [77]. Notably, LDLR expression was altered across high-grade cell lines and subtypes, indicating LDLR abnormalities as a potential ubiquitous metabolic target in pediatric and adult HGG patients with treatment potential in circumventing challenges presented by intra- and intertumoral heterogeneity [77]. LDLR is susceptible to lipid nanoparticle-based metabolic therapy, making this a clinically relevant finding with implications in clinical outcomes [77]. While mainly established in adult GBM cohorts, LDLR-targeted lipid nanoparticle therapies are being developed for targeted drug delivery [77]. Patients with the strongest potential response to LDLR-targeted therapy may rely on immunohistochemical quantification of LDLR expression to identify tumors with high cholesterol dependency [77]. Sphingolipids, particularly sphingosine-1-phosphate (S1P) and its G protein-coupled receptors, are highly involved in glioma cell proliferation and offer a metabolic vulnerability that can be exploited to inhibit tumor growth [78]. Researchers disrupted de novo sphingolipid synthesis using an RNA interference to silence serine palmitoyltransferase long chain base subunit 1, an enzyme that catalyzes the rate-limiting step of sphingolipid synthesis in glioma cells [78]. Inhibition of de novo sphingolipid synthesis in addition to blocking S1P receptor signaling and S1P export significantly reduced proliferation of p53 WT glioma cells through increased levels of p53 and p21 [78]. These findings reveal the pro-apoptotic effects of sphingolipid synthesis inhibition and introduce potential pharmacological targets for limiting glioma growth [78]. Several tumors, including adult GBM, are arginine auxotrophic due to an inability to synthesize or recycle intracellular arginine, leading to dependence on extracellular arginine [79]. To evaluate its relevance in pediatric brain tumors, researchers used immunohistochemical staining of key arginine pathway enzymes across tumor tissue microarrays from pHGGs, pLGGs, medulloblastomas, and ependymomas [79]. Researchers quantified consistent deficiencies in arginine recycling enzymes and increased expression of the arginine transporter SLC7A1 [79]. The urea cycle is the only metabolic pathway for endogenous arginine synthesis, meaning tumors that are deficient in one or more urea cycle enzymes, a key characteristic of pediatric brain tumors, must rely on external sources of arginine for survival [79]. pHGGs demonstrated the highest rates of deficiency in three key urea cycle enzymes, namely argininosuccinate synthetase, ornithine transcarbamylase, and argininosuccinate lyase, with deficiencies shown in 87.5%, 94%, and 79% of pHGG samples respectively [79]. Similarly high rates of argininosuccinate synthetase, ornithine transcarbamylase, and argininosuccinate lyase deficiencies were demonstrated across pLGGs and medulloblastomas, indicating urea cycle enzymes deficiencies as a key metabolic deviation across glioma grades and subtypes [79]. These findings provide evidence for arginine auxotrophy in pediatric brain tumors and offer arginine depletion as a new potential therapeutic strategy [79].

Metabolomics can also be used to optimize treatment regimens and uncover critical targets that may be used synergistically with current therapeutic methods. Enzymes critical for ketone metabolism–including 3-Hydroxybutyrate Dehydrogenase 1 (BDH1), 3-Oxoacid CoA-Transferase 1 (OXCT1), and Acetyl-CoA Acetyltransferase 1 (ACAT1)–were found to be downregulated in both pediatric and adult GBM, prompting investigation into potential defects in oxidative phosphorylation that may explain GBM’s reliance on glycolysis [80]. Using this knowledge, researchers tested an optimized therapeutic regime that combined both the glycolytic inhibitor 2-Deoxy-D-Glucose with ketone body acetoacetate, resulting in approximately 50% reduced viability in both pediatric and adult GBM and glioblastoma stem-like cell lines [80]. Notably, this effect was not observed with a combination of 2-Deoxy-D-Glucose and the ketone body β-hydroxybutyrate, indicating a specific cytotoxic effect of acetoacetate [80]. Mutations in *IDH1* are early events in gliomagenesis and result in an accumulation of the metabolite 2-Hydroxyglutarate (2-HG) [81]. In a first-in-human Phase 1 clinical trial (NCT02381886), an investigational mutant-selective IDH1 inhibitor drug (IDH305) was evaluated in 8 *IDH*-mutant glioma patients classified from WHO grades I–IV [81]. Using 3D MRS imaging, researchers observed a 70% reduction in 2-HG levels following one week of treatment, supporting the use of 2-HG as a biomarker to assess target inhibition and treatment response [81]. A prominent subtype of DMG, known as DMG-H3K27M, harbors a driver H3K27M mutation and is highly treatment resistant and prone to radio-resistance [3,82]. Researchers investigated the metabolomics of untreated and irradiated DMG-H3K27M tumors in cell lines, mouse models, and available patient data and uncovered a high reliance on purine synthesis through both de novo purine synthesis and purine salvage pathways [82]. To disrupt this, researchers inhibited de novo guanylate synthesis using mycophenolic acid and targeted guanylate salvage by silencing *hypoxanthine-guanine phosphoribosyltransferase* [82]. Cells treated with mycophenolic acid and radiotherapy experienced extended survival with eventual recurrence, while cells with *hypoxanthine-guanine phosphoribosyltransferase* knockdown and radiotherapy led to markedly prolonged survival and multiple complete responses [82]. These findings uncover crucial metabolic adaptations within DMG-H3K27M tumors and suggest that inhibiting purine salvage may be a promising strategy to overcome radiotherapy resistance [82]. Together, these findings highlight the power of metabolomic profiling in uncovering tumor-specific metabolic abnormalities that may guide therapeutic development for pediatric and adult brain tumors.

### 4.2. Combination Therapy Design and Precision Medicine

#### 4.2.1. Glycolytic Inhibitors

Tumor cells have been demonstrated to be highly reliant on glycolysis due to defects in ketone catabolism and oxidative phosphorylation [80]. Metabolomic profiling of pediatric glioma cells has allowed for the development of therapeutic protocols that exploit the tumor cells’ high reliance on glycolysis. Glycolytic inhibitors have been applied on their own or in combination with other therapies to explore their potential additive anti-tumoral benefits [83]. Pyruvate dehydrogenase kinase (PDK), hexokinase II, and LDHA have been identified as potential targets of glycolytic inhibitor therapy [83,84,85]. Dichloroacetate (DCA), a PDK inhibitor, has shown to induce proliferation arrest in GBM cell lines and primary human GBM cells [83]. Following successful completion of a Phase I clinical trial, an active Phase II clinical trial (NCT05120284) is investigating the use of oral DCA plus surgical resection in patients with recurrent GBM [84]. DCA has been used in combination with metformin, a widely used oral drug that inhibits complex I of the mitochondrial electron transport chain (ETC) and impairs tumor cells’ ability to perform oxidative phosphorylation [85]. DCA/metformin combination therapy demonstrated stronger anti-tumor effects, inducing cell death and downregulating key glycolytic enzymes including hexokinase II, LDHA, and enolase [85]. In a Phase II clinical trial (NCT02780024), metformin in conjunction with radiotherapy and temozolomide was shown to increase median survival time and progression-free survival time in adult GBM patients [86]. While DCA has been proposed as a treatment in conjunction with radiotherapy and temozolomide, the clinical trial was withdrawn before participant enrollment [87]. Clinical trial data has demonstrated potential side effects of glycolytic inhibitor therapies, including low incidence of distal paresthesia, although both DCA and metformin have shown to be generally tolerated at therapeutic dosing [84,86]. In a recent study using blood-based exosomes, metformin and cytoplasmic phospholipase A2, an intracellular enzyme that releases arachidonic acid, were demonstrated to selectively target GBM cells to impair mitochondrial energy metabolism and reduce tumor growth [88]. Ketoconazole and posaconazole, antifungal medications, have been shown to specifically target hexokinase II in patient-derived GSCs, cell lines, and mouse models of GBM [89]. While clinical trials in pediatric patients remain limited, relevant clinical trials in adult GBM and glioma patients present compelling evidence towards trialing glycolytic inhibitors in pediatric patients (Table 2).

#### 4.2.2. Amino Acid Metabolism Inhibitors

**Figure 3:**
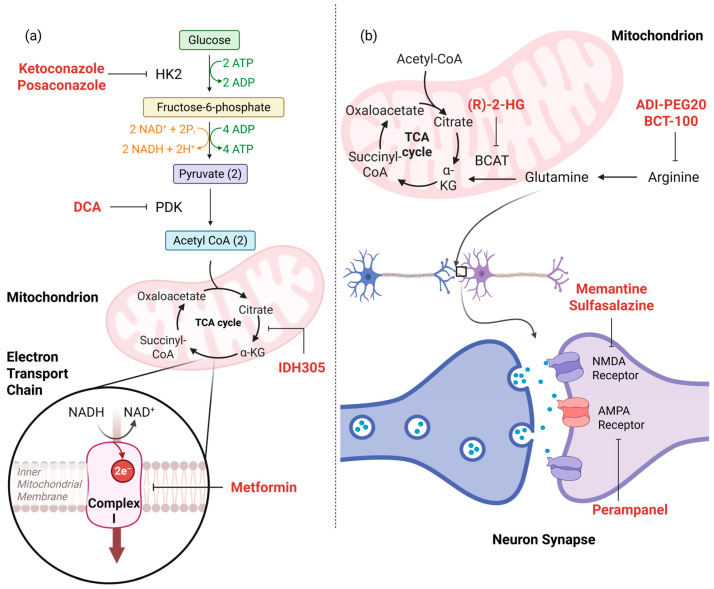
Metabolism-directed therapeutic approaches in glioma. (**a**) Glycolytic inhibitors. (**b**) Amino acid metabolism inhibitors. Ketoconazole and posaconazole, antifungal medications, inhibit hexokinase 2 (HK2), the enzyme necessary to initiate glycolysis by phosphorylating glucose. Dichloroacetate (DCA) is an inhibitor of pyruvate dehydrogenase kinase, resulting in induction of proliferation arrest. IDH305, a brain-penetrant *isocitrate dehydrogenase 1* (*IDH1*) inhibitor, blocks the ability of *IDH1* to convert isocitrate to α-ketoglutarate (α-KG), targeting the tumor cells’ tricarboxylic acid (TCA) cycle. Metformin, an oral drug that inhibits complex I of the electron transport chain (ETC), impairs tumor cells’ ability to perform oxidative phosphorylation. (R)-2-HG inhibits BCAT1 and BCAT2, halting glutamate production. Memantine and sulfasalazine, two experimental drugs, act as NMDA receptor antagonists; perampanel is an AMPA receptor antagonist. These antagonists work to impair glutamate reception in the brain, targeting tumor progression. ADI-PEG20 and BCT-100 deplete cells of arginine, blocking the ability of tumor cells to use arginine reservoirs as a source for glutamate and subsequently impairing glutamate biosynthesis. HK2, hexokinase 2; DCA, dichloroacetate; PDK, pyruvate dependent kinase; α-KG, α-ketoglutarate; TCA, tricarboxylic acid cycle; ETC, electron transport chain; ATP, adenosine triphosphate; ADP, adenosine diphosphate; NADH, nicotinamide adenine dinucleotide + hydrogen; IDH, isocitrate dehydrogenase, (R)-2-HG, R-2-hydroxyglutarate; BCAT, branched-chain amino acid aminotransferase; ADI-PEG20, pegylated arginine deiminase; BCT-100, pegylated recombinant human arginase; NMDA, N-methyl-D-aspartate; AMPA, α-amino-3-hydroxy-5-methyl-4-isoxazolepropionic acid. Created in BioRender. Kumar, K. (2026) (https://BioRender.com/kstg5xe, accessed on 14 March 2026).

Glioma cells exhibit marked abnormalities in amino acid metabolism, making this pathway a potential target for therapeutics (Figure 3). Inhibitors of amino acid metabolism target transaminases, glutaminase, or glutamate receptors to reduce tumor-driven excitotoxicity and tumor cell proliferation [90,91,92] (Table 3). (R)-2-HG inhibits the transaminases BCAT1 and BCAT2, enzymes necessary for glutamate production [90]. This impairment in glutamate biosynthesis makes glioma cells increasingly reliant on glutaminase for biosynthesis, rendering glioma cells vulnerable to oxidative stress [90]. Glutamate dependence is especially marked in *IDH*-mutant gliomas, presenting a strong potential target for therapeutics in *IDH*-mutant gliomas [90]. Recent studies have tested therapeutics targeting N-methyl-D-aspartate (NMDA) and AMPA receptors, which are glutamate receptors in the brain often implicated in tumor growth and tumor-related epilepsy [91,92]. Researchers demonstrated that perampanel, an AMPA receptor antagonist traditionally used as an anticonvulsant, showed systemic inhibitory effects on cell proliferation in patient-derived GBM and brain metastases [91]. In a pilot study of pediatric patients with primary CNS tumors, the NMDA receptor antagonist memantine was demonstrated to be a safe and well-tolerated addition to radiotherapy [93]. The study closed following the beginning of a Phase III clinical trial exploring the neurocognitive protective role of memantine in pediatric patients with CNS tumors receiving radiotherapy (NCT04939597) [93]. GLUGLIO (NCT05664464), a phase II trial of gabapentin, sulfasalazine, memantine, and chemoradiotherapy compared to chemoradiotherapy alone is actively exploring the efficacy and anti-glutamatergic effects of this combination therapy [92]. Presence of *IDH1/2* mutations, which increases glutamate dependency, or high levels of glutamate measured via MRS may aid in selection of patients for which amino acid metabolism therapies may be most effective [81]. Side effect profiles for gabapentin, sulfasalazine, memantine, and perampanel are well established. Gabapentin and perampanel most commonly cause adverse neurological symptoms, such as ataxia and dizziness [92,94]. Sulfasalazine is associated with hematologic, liver, and renal toxicity [92]. Toxicity from memantine is overall rare [92]. No results have been posted from the GUGLIO trial, limited evaluation of potential toxicity in patients.

Glioma and GBM cells have shown to be arginine auxotrophic, making these cells vulnerable to arginine inhibitors [95]. Arginine inhibitors function by targeting enzymes necessary for arginine synthesis, such as deiminases, or decreasing arginine availability [95,96]. In an intracranial animal model of human GBM, arginine depletion via pegylated arginine deiminase (ADI-PEG20) significantly reduced intracranial growth and extended survival time [95]. Further, the combination of ADI-PEG20 with the chemotherapeutic agent temozolomide demonstrated strongly enhanced anti-tumorigenic effects [95]. In the first systematic pediatric evaluation of arginine depletion (NCT03455140), researchers studied the safety and efficacy of pegylated recombinant human arginase (BCT-100) in 49 children with relapsed CNS cancers, including pHGGs [96]. Arginine depletion via BCT-100 was demonstrated to lead to sustained disease stability in highly aggressive refractory cancers, indicating a potential therapy for hard to treat tumors (Table 2) [96]. Arginine levels throughout the course of treatment were measured via patient blood samples, demonstrating adequate arginine depletion in both ADI-PEG20 and BCT-100 [96,97]. ADI-PEG20 has shown to be clinically well-tolerated, with major side effects observed in recipients including fatigue, constipation, and low-grade allergic reactions [97]. Incidence of grade 3 or higher toxicity is extremely rare in BCT-100, suggesting a low toxicity profile [96].

**Table 3:** Amino acid metabolism inhibitor clinical trials for pediatric and adult glioma or GBM patients.

NCT Number	Phase	Drug	Targets	Enrollment	Glioma Type	Status	Reference
NCT04939597	III	Memantine	NMDA receptor	192	Pediatric primary CNS tumors	Active, not recruiting	[93]
NCT05664464	Ib/II	Gabapentin, sulfasalazine, memantine, chemoradiotherapy	NMDA receptor, BCAT1, System Xc	120	GBM	Recruiting	[92]
NCT03455140	I/II	BCT-100	Arginine	49	Relapsed/refractory leukemia, neuroblastoma, sarcoma, and pHGGs	Completed	[96]
NCT04587830	II	ADI-PEG20	Arginine	100	GBM	Active, not recruiting	[97]

Exploiting the abnormalities in amino acid and glycolysis metabolism of pediatric and adult gliomas remains a promising active area of therapeutic exploration. Figure 3 summarizes the mechanism of action of glycolytic and amino acid metabolic therapies that are currently undergoing clinical trials in pediatric and adult brain tumor patients.

### 4.3. Real-Time Monitoring and Prognostic Applications

Recent advances in metabolomic techniques have enhanced diagnostic capabilities, monitoring of glioma progression, and prognostic prediction in patients [98]. LC-MS has been combined with machine learning methods to develop diagnostic panels for early detection of gliomas based on the presence of metabolites in plasma [98]. Analysis of 95 patients with WHO grade I-IV gliomas established that increased plasma levels of sphingomyelin and asparagine and decreased levels of taurine and choline are indicative of LGGs [98]. Similarly, LC-MS identified increased levels of tryptophan and methionine sulfoxide and depleted levels of cysteine, serine, and threonine correlated with a GBM diagnosis when compared to healthy controls [99]. In addition, in vivo proton MRS analysis determined that there is significantly elevated glycine levels in HGGs, suggesting enhanced serine–glycine–one-carbon metabolism in aggressive gliomas [100]. Short-echo time MRS analysis, a non-invasive imaging technique, was performed on 116 children with newly diagnosed brain tumors to investigate their tumor’s metabolic state [101]. This analysis demonstrated the applicability of glycine as a clinical biomarker of poor prognosis in pediatric gliomas, with increased levels of glycine correlating with higher tumor grade [101]. Alterations in the kynurenine pathway represent another example of metabolic dysregulation in gliomas. In a metabolomics study using ultra-high performance liquid chromatography-mass spectrometry, serum of GBMs showed increased levels of tryptophan and methionine sulfoxide, demonstrating enhanced tryptophan catabolism via indoleamine 2,3-dioxygenase activity [99]. These metabolic shifts are known to activate the aryl hydrocarbon receptor and suppress T cell activity, promoting an immunosuppressive TME [99]. Similarly, LC–MS and MRS have consistently detected elevated kynurenine and glutamine in glioma patient serum and cerebrospinal fluid (CSF) [102]. These findings reinforce the role of amino acid metabolism in avoiding immune-mediated targeting, detectable by real-time metabolomic assays. 

A broad range of studies applying MRS and MS to track treatment responses noted that changes in alternate metabolic levels, including lactate, choline, and 2-HG, often precede radiographic evidence of tumor progression or regression [103]. Elevated choline and lactate, detectable by MRS, have been linked to active tumor proliferation and hypoxia, whereas declining levels may reflect therapeutic response [103]. Similarly, glycine detection via echo-time-averaged ^1^H MRS provides a real-time window into the metabolic state of the tumor, potentially informing clinical decisions without the need for invasive biopsy [100]. Advanced computational techniques can be used to enhance the utility of metabolomics. Integrating machine learning with liquid chromatography-tandem mass spectrometry allowed for classification of glioma subtypes and grades via plasma-derived metabolic signatures [98]. This approach illustrates how machine learning can adaptively refine diagnostic and prognostic models based on metabolic input, enabling real-time, personalized surveillance of glioma progression [98]. Metabolomic-based assays can also be applied to demonstrate spatial heterogeneity in tumor metabolism. Orbitrap secondary ion mass spectrometry and liquid extraction surface analysis–tandem mass spectrometry has been used to map metabolites in human GBM tissue [104]. This enabled visualization of lipid and amino acid gradients across tumors, demonstrating intratumoral metabolic diversity that may influence invasion, hypoxia, and local immune suppression [104]. It has further been demonstrated that the spatial distribution of metabolic gene expression varies between LGGs and HGGs. HGGs are demonstrated to have a global decrease in mitochondrial-related genes and an upregulation of glycolysis-related genes in comparison to LGGs, especially in the rapidly proliferating regions of the glioma [105]. These findings could be applied to support the integration of spatial metabolomics into real-time intraoperative diagnostics and precision medicine-based treatments. Table 4 summarizes proposed biomarkers of glioma subtypes described in this review.

## 5. Discussion

Despite aggressive multimodal therapy, both pediatric and adult HGGs remain associated with poor clinical outcomes. Therapeutic insufficiency is driven by infiltrative growth and a highly heterogeneous cellular composition that enables glioma cells to dynamically adapt metabolism in response to radiotherapy and cytotoxic agents. In this context, metabolomics has emerged as a potential alternative approach to uncover glioma-specific metabolic abnormalities that may be therapeutically exploitable. Glioma metabolic reprogramming occurs in a constellation, with metabolic plasticity supporting proliferation, invasion, and therapeutic resistance. Metabolic plasticity in cancer cells that do not succumb to treatment has allowed surviving cancer cells to develop metabolic abnormalities that drive treatment resistance [68]. Metabolomic analyses across pediatric and adult gliomas have demonstrated that gliomas have distinct metabolic characteristics beyond the canonical Warburg effect. While enhanced aerobic glycolysis remains a hallmark of glioma biology, alterations in lipid and amino acid metabolism are demonstrated to be critical to glioma progression and invasion. Specific analyses suggest that these vulnerabilities differ primarily by tumor subtype, molecular driver, and biological context, with age serving as an important modifier rather than a standalone classifier. The identification of these metabolic aberrances has important therapeutic implications. Metabolic changes in gliomas are often due to oncogene-driven changes, marking metabolic therapies as a potential target for glioma cells that may spare surrounding healthy cells. This distinction is particularly important for pHGGs and HGGs, where conventional therapies are largely ineffective and disease is particularly infiltrative and diffuse. The reviewed studies across metabolomics suggest that metabolic plasticity underlies both therapy resistance and tumor proliferation. Glioma cells exhibit glycolytic flux and can actively shift between glycolytic, oxidative, and amino acid-dependent metabolic states based on nutrient availability, therapy-related stress, or other factors within the TME. Metabolic plasticity complicates therapeutic strategies but also represents therapeutic potential. Gliomas rely on distinct metabolic aberrations to support proliferation, and these compensatory pathways can potentially be targeted with metabolic-based therapies. Clinical and pre-clinical studies targeting glycolytic pathways, glutamate production and absorption, and arginine metabolism demonstrate that metabolic therapies have anti-tumor effects and, in some cases, enhance sensitivity to radiotherapy and cytotoxic agents [83,84,85,86,87,92,93,96,97]. However, therapeutic response is heterogeneous and subtype-dependent, underscoring the need for biomarker-driven patient stratification to inform metabolic therapies.

Despite advancements in the field of glioma metabolomics, several limitations remain in the current literature. Pediatric-specific preclinical and clinical studies remain comparatively sparse, making it difficult to evaluate the efficacy and safety of metabolic inhibitors in the pediatric population. As a result, the field continues to rely heavily on adult data and, at times, extrapolation from non-glioma tumors. Importantly, metabolic differences are best interpreted according to tumor entity, molecular driver, and microenvironmental context rather than age alone. For example, DMG H3K27M-altered tumors exhibit a biologically distinct and highly treatment-resistant state shaped by epigenetic dysregulation, while IDH1/2-mutant gliomas are defined by accumulation of D2-HG and associated alterations in glutamate, phospholipid, and energy metabolism. Although *IDH1/2* mutations are present across age groups, they are predominantly found in the adult population with fewer pediatric occurrences recorded in the literature. These distinctions highlight the need for entity-specific study design and biomarker-driven therapeutic development. Another critical limitation is the inherent metabolic heterogeneity of gliomas. A single biopsy may reveal metabolic signatures of a subpopulation of glioma cells, but it fails to capture the metabolic profile of the whole tumor. Metabolism varies by region, with key areas such as the necrotic core and invasive edge exhibiting hallmark differences in metabolism. Tumor cells exhibit metabolic plasticity and adapt metabolic output in response to changes in the TME and treatment strategies, complicating the snapshot provided by a single metabolic analysis. Tumor cells can be highly compartmentalized, with certain metabolic processes occurring in surrounding stromal and immune cells, as well as between intracellular and extracellular spaces. A snapshot metabolite measurement will not necessarily reflect the complexity and spatial heterogeneity within a glioma and its corresponding TME. Metabolomic profiles also vary greatly between patients, making intertumoral heterogeneity a complicating factor. Metabolomic analyses may overlook biologically relevant subpopulations of glioma cells that harbor metabolic abnormalities that drive tumor proliferation and invasion due to limitations in tumor samples. Limitations in metabolomic analyses due to intratumoral heterogeneity may be partially addressed by integrating metabolomics with complementary approaches such as single-cell RNA sequencing (scRNA-seq) or spatial transcriptomics, as these analyses can reveal metabolic pathway regulation and metabolic states at the single-cell level. scRNA-seq is an analytic technique to characterize gene expression across individual cells, and spatial transcriptomics maps gene expression within the tumor context [105]. Importantly, metabolomics refers to the measurement of metabolites within a sample, while transcriptomics refers to measurements of gene expression. Gene expression does not always perfectly correlate with levels of metabolites, making transcriptomic analyses best viewed as a complementary tool that can help contextualize metabolomic findings. A previous study utilizing scRNA-seq and spatial transcriptomics revealed alterations in pathways triggered in response to oxidative stress in both HGGs and LGGs, marking a novel perspective on stress responses across glioma subtypes [105]. Another study demonstrated key differences between two prognostic glioma subtypes using single-cell transcriptomics, with marked differences in glycolysis, fatty acid oxidation, and the pentose phosphate pathway that could be used to guide patient-specific therapies [106]. Further, spatial metabolomics can be used to map metabolic gradients within the tumor, identifying regions of metabolic patterns within a glioma. Spatial metabolomic analysis has demonstrated grade-specific and region-specific metabolic vulnerabilities and dependencies in grade II-IV gliomas, providing a potential resource to identify specific metabolic targets within glioma cells [107]. These metabolic analyses could aid in capturing the entire metabolic profile of the glioma. Spatial metabolomics also has the potential to reveal new metabolic abnormalities that have previously been overlooked or missed due to limitations in tissue biopsy. The identification of biomarkers and the baseline metabolic state may further help overcome difficulties in treatment presented by intertumoral heterogeneity. Identification of large metabolic subclasses, such as *IDH*-mutant status, and patient stratification within clinical trials may help identify which metabolic inhibitors are most effective against specific metabolic subclasses, informing therapeutic decision making. Difficulties remain in translating these metabolic targets into clinical practice due to hurdles in biomarker validation and drug delivery. While metabolomics identifies potential biomarkers of different tumor subtypes and grades, along with prognosis, validating these biomarkers across diverse clinical cohorts remains difficult due to the transient nature of metabolites and the lack of standardized sampling protocols. Furthermore, the inherent limiting nature of the blood–brain barrier remains a significant constraint in drug delivery to the CNS, including in delivery of metabolic inhibitors. Targeted metabolic inhibitors may fail to cross the blood–brain barrier in sufficient concentration to produce a therapeutic response within the tumor parenchyma. Metabolomic analyses are further complicated by a lack of consensus protocols or guidelines for sample preparation, data acquisition, and downstream analyses. Variability in tissue collection protocols, storage conditions, solvents, and analytical procedures introduce technical bias, making it difficult to distinguish biological difference from artifact. The lack of standardization complicates meta-analyses and integration of metabolomic data, hampering the establishment of robust metabolic biomarkers. Addressing these challenges will require the development of harmonized protocols and quality control standards to enable meaningful clinical interpretations.

A critical avenue of future research lies in the integration of metabolic inhibitors with traditional treatment therapies, including surgery, radiotherapy, chemotherapy. Reviewed evidence suggests that metabolic targeting is more likely to be maximally effective in combination therapy due to the pronounced metabolic plasticity of glioma cells [108]. Combination therapies to exploit therapy-induced stress may offer greater anti-tumor effects than monotherapy alone. Early studies of dual metabolic targeting with DCA and metformin have demonstrated a synergistic anti-tumor effect of combining these glycolytic inhibitors [108]. This suggests that simultaneous inhibition of glycolysis limits the tumor cells’ ability to rely on compensatory pathways, leading to enhanced anti-proliferative effects. The study demonstrated that DCA and metformin synergistically suppress cell proliferation only at high doses, raising a potential concern about dosing [108]. Sufficient dosing to produce anti-proliferative effects could contribute to increased side effects in a combination therapy context, including gastrointestinal discomfort and peripheral neuropathy [108]. Future studies should seek to elucidate dosing of metabolic inhibitors that produces anti-proliferative effects while minimizing side effects. Nonetheless, the strongest anti-tumor effects have been demonstrated with metabolic inhibitors used in conjunction with traditional therapies. Metformin in combination with radiotherapy and temozolomide demonstrates the strongest anti-proliferative effects and aids in chemoresistance [86]. This result adds to the growing body of evidence that metformin, and potentially other metabolic targeted therapies, serve as sensitizing agents for traditional therapeutic strategies. These data support further investigation of combination metabolic inhibitor and standard therapy approaches, as well as dual metabolic targeting approaches, to determine which drug combinations produce the strongest anti-tumor effects in specific glioma subtypes and age cohorts.

Development of advanced diagnostics, such as MRS, will permit the use of non-invasive real-time metabolic analyses in research and clinical settings. Real-time analytics is particularly valuable in understanding pHGG biology, where surgical sampling is often unsafe due to diffuse infiltration of tumor cells. MRS and other advanced diagnostics can capture intratumoral metabolic heterogeneity and metabolic flux in response to treatment, potentially allowing for real-time treatment monitoring and early detection of therapeutic resistance. The continued refinement of advanced metabolic diagnostics has the potential to enable longitudinal metabolic profiling, facilitating precision medicine approaches that allow real-time changes in treatment strategies in response to tumor cells’ metabolic flux. Shifting to precision medicine approaches would allow a change from standardized therapeutic strategies to treating each patients’ tumor as a unique, evolving biosystem. Advanced metabolomics could be paired with proteomics and genomics to capture the full complexity of glioma biology in an integrated multi-omics approach. Genomic analyses can potentially inform the identification of upstream regulatory changes that directly influence metabolic pathway preferences and vulnerabilities. Proteomics can bridge the gap between genomic and metabolomic analyses by identifying changes in enzyme expression or signaling pathway activity involved in metabolic aberrations. A multi-omics approach could potentially enable the identification of vulnerabilities across glioma subtypes, improving biomarker discovery and therapeutic target analyses. Combining metabolomics with genomics and proteomics provides a more comprehensive understanding of glioma cell biology, supporting the development of the field of metabolic-based therapies. By integrating multi-omic data with real-time monitoring, clinicians can tailor interventions to specific biochemical signatures within an individual tumor, ensuring that therapy is not only targeted to the initial diagnosis but is adapted to match the individual glioma’s unique physiological response to treatment. This has the potential to minimize unnecessary toxicity to ineffective therapeutics and maximize targeted therapeutic efficacy by ensuring that selected drugs target the unique vulnerabilities of each patients’ tumor.

Despite these limitations, this review advances the current understanding of metabolomic profiling in pediatric and adult gliomas and highlights the emerging clinical potential of metabolomics-driven therapeutic strategies. To our knowledge, this manuscript represents the first comprehensive synthesis of metabolomic profiling and metabolism-targeted therapeutic approaches across both pediatric and adult gliomas. Metabolomic signatures offer an opportunity to refine tumor classification, identify predictive biomarkers, and improve prognostic assessment. Importantly, integrating metabolically targeted therapies with conventional treatment strategies, including pediatric-specific therapeutic approaches, may reveal previously unrecognized metabolic vulnerabilities and enable more precise tumor-specific interventions. Continued advances in metabolomic technologies and metabolic targeting hold promise for the development of personalized treatment strategies that may ultimately improve progression-free and OS in patients with glioma. Collectively, the growing integration of metabolomic analyses with therapeutic development represents a promising avenue to enhance treatment efficacy and clinical outcomes for both pediatric and adult glioma patients.

## Figures and Tables

**Table 2:** Glycolytic inhibitor clinical trials for pediatric and adult glioma patients.

NCT Number	Phase	Drug	Targets	Enrollment	Glioma Type	Status	Reference
NCT02381886	I	IDH305	*IDH1*	166	*IDH*-mutant glioma	Active, not recruiting	[81]
NCT05120284	IIA	DCA	PDK	40	GBM	Active, not recruiting	[84]
NCT02780024	II	Metformin	ETC	50	GBM	Active, not recruiting	[86]

**Table 4:** Proposed biomarkers of glioma subtype, grade, or prognosis.

Biomarker	Indication	Glioma Subtype
O6-methylguanine DNA methyltransferase	Improved OS	GBM
Downregulated BDH1, OXCT1, ACAT1	Reliance on glycolysis	Pediatric and adult GBM
Glycine	Poor prognosis	GBM, HGG
High choline/creatinine ratio	Severe tumor infiltration	HGG
Elevated taurine, phenylalanine, tyrosine	Altered osmoregulation	HGG
LDLR upregulation	Tumor proliferation	HGG and pHGG
L1 cell adhesion molecule, SOX2, Nanog	High-grade disease, metabolic plasticity	pHGG
Elevated choline	Tumor proliferation	pHGG
Elevated lactate, alanine	High-grade disease	pHGG
LPE, low NAA/metabolite ratio	Better prognosis	LGG
Elevated carnitine	Better prognosis	*IDH1*-mutant glioma
2-HG	Target inhibition and treatment response	*IDH1*-mutant glioma
Elevated choline, lactate	Active tumor proliferation	*IDH1*-mutant glioma
D2-HG	Seizure activity	*IDH1*-mutant glioma
Low glucose	Increased glycolysis	Medulloblastoma
Elevated phenylalanine, tyrosine, tryptophan	Aberrations in amino acid metabolism	Medulloblastoma
Elevated taurine, glycine, CCMs	Glioma cell invasion	Metastatic medulloblastoma

## Data Availability

No new data were created or analyzed in this study. Data sharing is not applicable to this article.

## Abbreviations

The following abbreviations are used in this manuscript:

2-HG	2-hydroxyglutarate
α-KG	α-ketoglutarate
ACAT1	Acetyl-CoA acetyltransferase 1
AMPA	α-amino-3-hydroxy-5-methyl-4-isoxazolepropionic acid
ATP	Adenosine triphosphate
BDH1	β-hydroxybutyrate dehydrogenase 1
BDNF	Brain-derived neurotrophic factor
CCM	Choline-containing metabolite
CNS	Central nervous system
CSF	Cerebrospinal fluid
D2-HG	D-2-hydroxyglutarate
DCA	Dichloroacetate
DMG	Diffuse midline glioma
ETC	Electron transport chain
GABA	Gamma-aminobutyric acid
GBM	Glioblastoma
GSC	Glioma stem cell
HGG	High-grade glioma
IDH	Isocitrate dehydrogenase
LC-MS	Liquid Chromatography-Mass Spectrometry
LDHA	Lactate Dehydrogenase A
LDLR	Low-density lipoprotein receptor
LGG	Low-grade glioma
LPE	Lysophosphatidylethanolamine
LPS	Lipopolysaccharide
MDH2	Malate dehydrogenase 2
MRS	Magnetic resonance spectroscopy
MS	Mass spectrometry
MYC	Myelocytomatosis
NAA	N-acetylaspartate
NK	Natural killer cell
NMDA	N-methyl-D-aspartate
NMR	Nuclear magnetic resonance
OPC	Oligodendrocyte precursor cell
OS	Overall survival
OXCT1	3-oxoacid CoA-transferase 1
PDHGG	Pediatric-type diffuse high-grade glioma
PDK	Pyruvate dehydrogenase kinase
S1P	Sphingosine-1-phosphate
scRNA-seq	Single-cell RNA sequencing
SHH	Sonic hedgehog
SMO	Smoothened mouse model
TAM	Tumor-associated macrophage
TCA	Tricarboxylic acid cycle
TME	Tumor microenvironment
WHO	World health organization
WT	Wild type
